# VPS33A and VPS18 orchestrate porcine epidemic diarrhea virus replication by modulating autophagic flux

**DOI:** 10.1080/21505594.2026.2707880

**Published:** 2026-07-31

**Authors:** Liang Guo, Jin Li, Zhuolin Hao, Kexin Wang, Yikai Shi, Fei Gao, Xuguang Du, Sen Wu

**Affiliations:** a Sanya Institute of China Agricultural University, Sanya, China; b Frontiers Science Center for Molecular Design Breeding (MOE), State Key Laboratory of Animal Biotech Breeding, College of Biological Sciences, China Agricultural University, Beijing, China

**Keywords:** Complete autophagy, nsp3, nsp4, PEDV, VPS18, VPS33A

## Abstract

Completion of autophagy through autophagosome-lysosome fusion represents a critical regulatory checkpoint that is frequently manipulated during viral infection, however, how coronaviruses exploit this terminal step remains incompletely understood. Porcine epidemic diarrhea virus (PEDV), a highly pathogenic alphacoronavirus, has been reported to induce autophagy, but whether and how complete autophagic flux contributes to viral replication is unclear. Here, using a genome-wide CRISPR/Cas9 knockout screen in Vero cells, we identify the HOPS complex subunits VPS33A and VPS18 as essential host factors for PEDV infection. Genetic ablation of either VPS33A or VPS18 profoundly suppresses viral progeny production and arrests autophagic flux at the autophagosome stage, demonstrating that PEDV replication is associated with HOPS-mediated autolysosome formation. Domain-mapping analyses further reveal that the structural integrity of VPS33A and the α-solenoid plus RING domains of VPS18 are indispensable for both autophagosome-lysosome fusion and efficient viral replication. Mechanistically, we show that PEDV non-structural proteins nsp3 and nsp4 physically interact with VPS33A and VPS18. While either protein alone initiates autophagosome formation, their coordinated action contributes to drive complete autophagy in a HOPS-dependent manner. Collectively, our findings uncover a coronavirus strategy that actively promotes autophagic completion through VPS33A and VPS18, two core subunits of the HOPS complex, to support replication, thereby establishing autolysosome formation as a critical host process exploited by PEDV.

## Introduction

Porcine epidemic diarrhea virus (PEDV) is a highly contagious pathogen that causes watery diarrhea, vomiting, and rapid dehydration in suckling piglets, with mortality rates approaching 100% in neonates [1,2]. Phylogenetic analysis of the spike (S) gene has classified PEDV strains into three major genotypes: classical G1, variant G2, and S-INDEL. The G1 genotype was first identified in Europe during the 1970s [3,4]. In 2010, a highly virulent G2 strain emerged in China and rapidly spread to North America, Europe, and several Asian countries [5]. Subsequently, the G2 genotype has diversified into three sub-lineages: G2a, G2b, and G2c, with recent epidemiological surveys indicating a marked increase in outbreaks associated with G2c [6,7]. Although vaccination remains the primary strategy for PEDV control, continual viral evolution through antigenic drift and recombination poses significant challenges to vaccine efficacy. Consequently, there is an urgent need to identify novel antiviral targets by elucidating host factors essential for PEDV infection. Previous genome-wide CRISPR knockout (KO) screens performed in cell lines such as Huh7, LLC-PK1, Vero E6, HEK293T, IPEC-J2, and IPI-2I have identified several host dependency factors, including ST3GAL4, IFITM family proteins, RPSA, TRIM2, SLC35A1, PKCθ, and YIPF5, which are critical for PEDV infection [8–13]. In the present study, we constructed a comprehensive genome-wide CRISPR KO library in Vero cells and performed a PEDV challenge screen. Our screening identified two host factors not previously implicated in PEDV infection, VPS33A and VPS18, both of which are core components of the homotypic fusion and protein sorting (HOPS) complex.

HOPS is a key multi-protein assembly in intracellular vesicle transport that orchestrates membrane flow and vesicle fusion. By mediating Rab7-dependent fusion of late endosomes with lysosomes, it safeguards endosomal identity, and without it the entire system collapses into disarray [14,15]. The HOPS complex also supervises autophagosome maturation. Lysosomal RAB39A, once activated by C9orf72, recruits HOPS-4 (VPS41-VPS16-VPS18-VPS33A), while GTP-bound RAB2 on autophagosomes recruits HOPS-2 (VPS39-VPS11). These sub-complexes then zip together into HOPS-6, tethering the autophagosome to the lysosome and licensing the final STX17-SNAP29-VAMP8 SNARE-driven fusion [16,17]. Consequently, HOPS acts as a master regulator of both endocytic and autophagic membrane traffic in eukaryotic cells, and its subunits have emerged as critical host cofactors for numerous viruses. Genome-wide haploid screens reveal that loss of any one of the six HOPS subunits confers robust resistance to Ebola virus entry [18], and depletion of VPS18 or its cofactor Mon2 severely impairs HIV-1 particle production [19]. Conversely, viruses can also target HOPS to sabotage autophagic flux. SARS-CoV-2 ORF7a initiates autophagy but blocks completion by proteasomal degradation of SNAP29 [20–22], whereas ORF3a sequesters VPS39, disrupting HOPS-RAB7-STX17 interactions and arresting autolysosome formation [23,24]. Whether PEDV exploits or antagonizes this HOPS-mediated autophagy checkpoint remains largely unresolved.

Macroautophagy (hereafter referred to as autophagy) is a conserved catabolic process that begins with ULK1-complex activation, proceeds through PI3K-driven nucleation and ATG-mediated elongation and closure of the phagophore, and culminates in fusion with lysosomes to form autolysosomes, where lysosomal enzymes degrade and recycle cargo to preserve cellular homeostasis [25,26]. Accumulating evidence portrays autophagy as a double-edged sword in viral infection: it can eliminate invading pathogens yet is frequently hijacked to support viral replication [27–29]. Host cells deploy specific factors such as HNRNPA1, POLM, BTG3, and ZNF16 to harness autophagy for the degradation of viral proteins and thereby restrict PEDV replication [30–33]. Specifically, HNRNPA1 activates the MARCHF8-CALCOCO2/NDP52-autophagosome pathway to degrade the viral nucleocapsid (N) protein [30]. POLM interacts with MARCH8 and p62 to facilitate the autophagic degradation of PEDV N, S2, and M proteins [31]. BTG3 targets the S2 subunit of the spike (S) protein for degradation through both autophagy and proteasome pathways by recruiting the cargo receptor NDP52 and the E3 ubiquitin ligase MARCHF8 [32]. ZNF16 recruits the E3 ubiquitin ligase STUB1 to promote S1 ubiquitination, which is subsequently recognized by the cargo receptor Tollip for translocation to autolysosomes, ultimately leading to S1 degradation [33]. Conversely, multiple studies show that PEDV infection induces autophagy and this induction benefits viral progeny output [34–36]. Among viral products, ORF3 and non-structural protein (nsp) 6 are the established drivers: ORF3 triggers ER stress via the GRP78-PERK-eIF2α axis, whereas nsp6 dampens the PI3K/Akt/mTOR cascade to initiate autophagosome formation [35,37]. However, whether nsp3 and nsp4 modulate autophagy remains unexplored.

In this study, we constructed a genome-wide CRISPR knockout Vero cell library and screened for host genes essential for PEDV replication. Among the top hits were VPS33A and VPS18, core subunits of the HOPS complex. Independent VPS33A or VPS18 KO cell lines produced markedly fewer progeny viruses, demonstrating that both proteins are required during the replication stage of the viral life cycle. Mechanistic analyses revealed that loss of either protein impairs autolysosome formation and suppresses PEDV replication. The integrity of VPS33A and the α-solenoid plus RING domains of VPS18 both contribute to efficient autolysosome biogenesis and optimal viral replication. PEDV nsp3 and nsp4 interacted with VPS33A/VPS18 and contributed to complete autophagy. Collectively, these data identify VPS33A and VPS18 as promising therapeutic targets for the prevention and treatment of PEDV infection.

## Results

### Genome-wide CRISPR/Cas9 screen identifies VPS33A and VPS18, two core subunits of the HOPS complex, as essential host factors for PEDV infection

**Figure 1.**
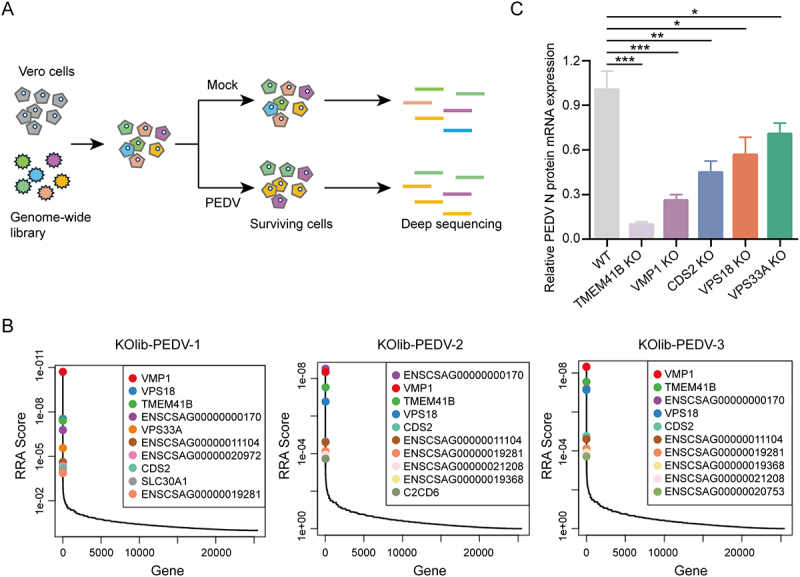
Genome-wide CRISPR/Cas9 screen identifies HOPS complex subunits VPS33A and VPS18 as essential host factors for PEDV infection. (A) Schematic of the screening workflow. The African green monkey genome-scale CRISPR/Cas9 knockout library transduced into Vero cells was challenged with PEDV (MOI = 0.001, 60 hpi). Surviving cells were expanded, genomic DNA was extracted, sgRNA sequences were amplified by PCR, and subsequently were subjected to deep sequencing. (B) Gene-level robust rank aggregation (RRA) scores calculated with MAGeCK for three independent screens (KOlib-PEDV-1, −2, −3). (C) Validation of candidate genes. The pooled knockout Vero cells were infected with PEDV (MOI = 0.01, 24 hpi) and viral N mRNA levels were quantified by RT-qPCR. Abbreviations: WT, wild type; KO, knockout; MOI, multiplicity of infection; hpi, hours post-infection; N, nucleocapsid. Data are representative of at least three independent experiments and are presented as mean ± SD. *p < 0.05; **p < 0.01; ***p < 0.001. *p*-values were determined by two-sided Student’s t-test.

To systematically identify host factors required for PEDV infection, we performed a genome-wide CRISPR/Cas9 knockout screen in Vero cells using the GeCKO v2 library. The pooled mutant cells were infected with PEDV strain CV777 at a multiplicity of infection (MOI) of 0.001 and cultured until complete cytopathic effect was observed in wild type (WT) Vero cells. Surviving cells were harvested, and single-guide RNAs (sgRNAs) were amplified and quantified by deep sequencing (Figure 1(A)).

Gene-level robust ranking aggregation (RRA) scores were calculated with the MAGeCK software package, and the top 10 ranked genes were visualized (Figure 1(B), Table S1). Across three independent screens, the well-established coronavirus dependency factors TMEM41B and VMP1 were reproducibly identified [38–40]. Notably, two core subunits of the HOPS complex, VPS33A and VPS18, ranked among the top hits (Figure 1(B)).

Five high-priority genes (TMEM41B, VMP1, CDS2, VPS18, and VPS33A) were selected for validation. Pooled knockout cell lines were generated (Figure S1) and challenged with PEDV. RT-qPCR revealed a marked reduction in PEDV N mRNA levels upon individual knockout of each candidate gene (Figure 1(C)), confirming their critical roles in PEDV infection. However, these results represented only preliminary validation, and the major conclusions are based on subsequent clonal biallelic knockout, rescue, and overexpression experiments. Given the established role of the HOPS complex in autophagosome-lysosome fusion, we focused on VPS33A and VPS18 for further validation.

### HOPS complex subunits VPS33A and VPS18 are critical host factors for PEDV replication

**Figure 2.**
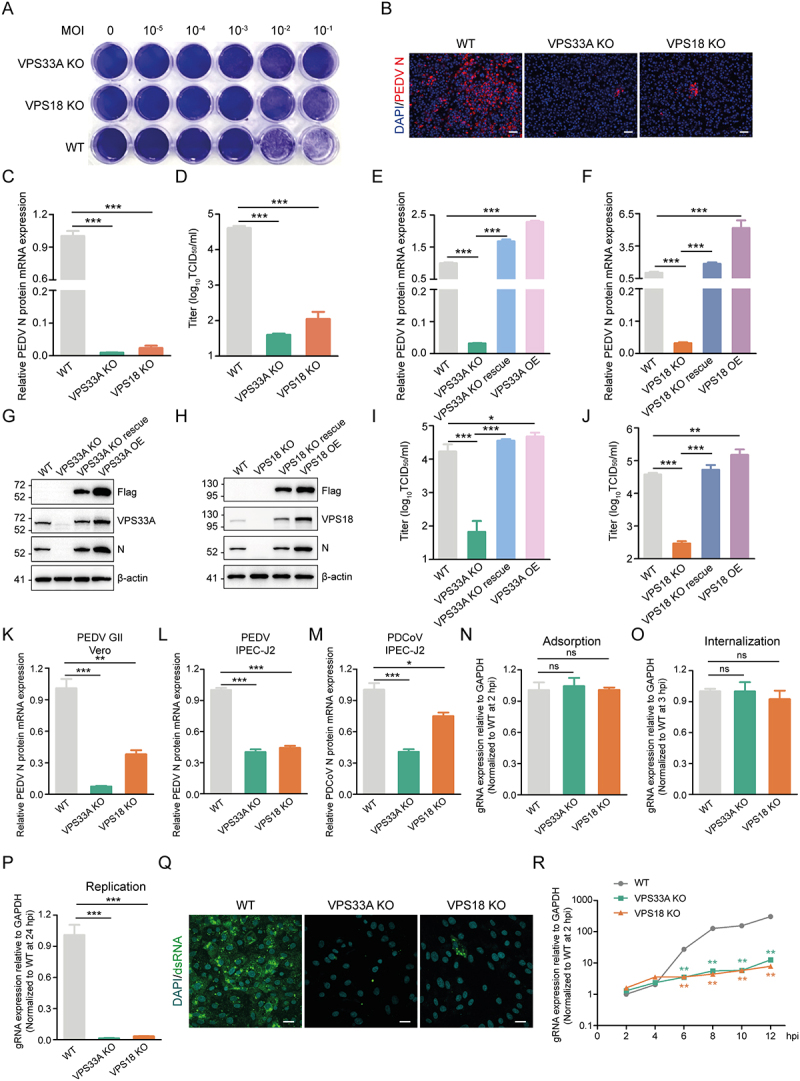
HOPS complex subunits VPS33A and VPS18 are essential host factors required for PEDV replication. (A) Plaque assays. WT, VPS33A KO, and VPS18 KO Vero cells were seeded into 24-well culture plates and infected with serial 10-fold dilutions of PEDV (MOI = 0, 10^−5^, 10^−4^, 10^−3^, 10^−2^, 10^−1^). Plaques were visualised with 1% crystal violet. (B) Immunofluorescence of PEDV N protein in WT, VPS33A KO, and VPS18 KO Vero cells (MOI = 0.01, 24 hpi). Scale bars, 200 μm. (C) RT-qPCR quantification of the relative N mRNA level in WT, VPS33A KO, and VPS18 KO Vero cells (MOI = 0.01, 24 hpi). (D) TCID_50_ of culture supernatants from WT, VPS33A KO, and VPS18 KO Vero cells (MOI = 0.01, 24 hpi). (E, F) RT-qPCR of the relative N mRNA in rescue and overexpression (OE) Vero lines for VPS33A (E) or VPS18 (F) (MOI = 0.01, 24 hpi). (G, H) Western blot assays for detecting the Flag tag, VPS33A (G) or VPS18 (H), PEDV N and β-actin in the same lines as (E, F). (I, J) TCID_50_ of culture supernatants from rescue and OE Vero lines for VPS33A (I) or VPS18 (J) (MOI = 0.01, 24 hpi). (K) RT-qPCR of the relative N mRNA level in WT, VPS33A KO, and VPS18 KO Vero cells challenged with PEDV LJX strain (subtype GII) (MOI = 0.01, 24 hpi). (L, M) RT-qPCR of the relative N mRNA level in WT, VPS33A KO, and VPS18 KO IPEC-J2 cells challenged with PEDV (L) (MOI = 0.1, 48 hpi) or PDCoV (M) (MOI = 0.01, 48 hpi). (N) Evaluation of PEDV adsorption activity on WT, VPS33A KO, and VPS18 KO Vero cells by RT-qPCR. Cells were infected with PEDV (MOI = 10) at 4°C for 2 h. (O) Assessment of PEDV internalization stage in WT, VPS33A KO, and VPS18 KO Vero cells by RT-qPCR. Cells were infected with PEDV (MOI = 5) at 4°C for 2 h and transferred to 37°C for 1 h. (P) Analysis of PEDV replication activity in WT, VPS33A KO, and VPS18 KO Vero cells by RT-qPCR. Cells were infected with PEDV (MOI = 0.01) at 37°C for 24 h. (Q) Analysis of PEDV replication activity in WT, VPS33A KO, and VPS18 KO Vero cells by immunofluorescence staining. Cells were infected with PEDV (MOI = 0.01) at 37°C for 24 h. (R) Viral growth kinetics within 12 h in WT, VPS33A KO, and VPS18 KO Vero cells. Abbreviations: WT, wild type; KO, knockout; MOI, multiplicity of infection; hpi, hours post-infection; N, nucleocapsid; ns, no significant. Data are representative of at least three independent experiments and are presented as mean ± SD. *p < 0.05; **p < 0.01; ***p < 0.001. *p*-values were determined by two-sided Student’s t-test.

To rigorously confirm the roles of HOPS complex subunits VPS33A and VPS18 in PEDV infection, we isolated monoclonal cell lines harboring homozygous deletions of VPS33A or VPS18 from the respective pooled knockout populations. Sanger sequencing confirmed biallelic disruption in both knockout cell lines, and proliferation assays showed that loss of either gene had no appreciable effect on cell growth (Figure S2A, B). WT, VPS33A KO, and VPS18 KO Vero cells were challenged with PEDV CV777 strain (subtype GI). Plaque assays revealed that VPS33A KO or VPS18 KO Vero cells remained more viable than WT Vero cells after PEDV infection at MOIs of 0.1 and 0.01 (Figure 2(A)). Immunofluorescence and RT-qPCR analyses of PEDV N protein showed that knockout of VPS33A or VPS18 markedly reduced N protein expression (Figure 2(B,C)). Moreover, TCID_50_ assays demonstrated that either knockout significantly reduced viral titers (Figure 2(D)).

To further validate these findings, we generated rescue and overexpression cell lines for each protein, all of which were Flag-tagged. RT-qPCR, Western blot and TCID_50_ analyses confirmed that re-expression of VPS33A or VPS18 fully restored PEDV replication, and overexpression of either protein further increased viral replication compared with WT cells (Figure 2(E–J)). Next, we examined the effect of VPS33A or VPS18 knockout on replication of the PEDV LJX strain (subtype GII). RT-qPCR revealed that loss of either gene markedly attenuated GII viral N mRNA levels (Figure 2(K)). To exclude species-specific effects, we knocked out VPS33A or VPS18 in IPEC-J2 cell lines (Figure S2C). Both PEDV and PDCoV replicated poorly in these knockout cells (Figure 2(L,M)), indicating that VPS33A and VPS18 may be broadly essential for coronavirus infection.

To determine which step of the PEDV life cycle is affected by VPS33A or VPS18, we performed adsorption, internalization, and replication assays. For adsorption, WT, VPS33A KO, and VPS18 KO Vero cells were incubated with PEDV at 4°C for 1 h. RT-qPCR of cell-associated viral genomic RNA (gRNA) showed no significant differences between WT and KO Vero cells, indicating that VPS33A and VPS18 are dispensable for viral adsorption (Figure 2(N)). For internalization, cells were pre-bound with virus at 4°C for 2 h, then shifted to 37°C for 1 h to allow internalization. Intracellular viral gRNA levels were comparable between WT and KO Vero cells, demonstrating that VPS33A and VPS18 are also not required for virus internalization (Figure 2(O)). Having excluded roles in adsorption and internalization, we focused on replication. Cells were infected at MOI of 0.01 and harvested at 24 h post-infection (hpi). RT-qPCR revealed a marked reduction in PEDV gRNA in both VPS33A KO and VPS18 KO relative to WT Vero cells (Figure 2(P)). We also performed immunofluorescence staining to assess PEDV dsRNA levels in these cells, and the results showed that dsRNA was similarly decreased in VPS33A KO and VPS18 KO Vero cells (Figure 2(Q)). To pinpoint the critical time window, we performed a 12 h time-course sampling every 2 h. Viral gRNA levels remained similar up to 4 hpi, but from 6 hpi onward replication was significantly suppressed in KO Vero The hookup model of the HOPS complex cells, whereas WT cultures continued exponential growth (Figure 2(R)). Collectively, these data demonstrate that VPS33A and VPS18 are essential for efficient PEDV genome replication.

### VPS33A/VPS18 deficiency impairs viral replication and correlates with autolysosome formation

Previous studies have shown that the HOPS complex catalyzes the fusion of autophagosomes with lysosomes [16,17]. PEDV replication depends on completion of this autophagic flux, and chloroquine (CQ)-mediated blockade of the fusion suppresses viral propagation [34]. We therefore hypothesized that deletion of VPS33A or VPS18 would interrupt PEDV-induced autophagic flux and consequently inhibit viral replication. First, we confirmed that PEDV-induced autophagy requires viral replication and that CQ treatment reduces viral replication (Figure S3A, B).

**Figure 3.**
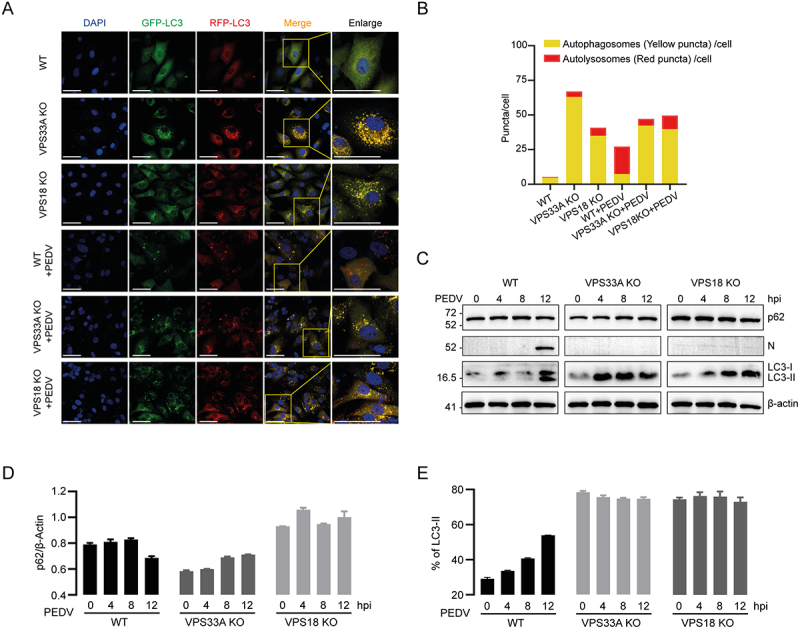
HOPS complex subunits VPS33A or VPS18 knockout impairs autolysosome formation and suppresses PEDV replication. (A) Confocal images of autophagosome and autolysosome formation in uninfected and PEDV-infected WT, VPS33A KO, and VPS18 KO Vero cells (MOI = 0.01, 24 hpi). Scale bars, 50 µm. (B) Quantification of autophagosomes (yellow) and autolysosomes (red) shown in (A). (C) Western blot analysis of p62, LC3, and PEDV N protein in WT, VPS33A KO, and VPS18 KO Vero cells after PEDV infection (MOI = 0.01). β-actin served as loading control. (D, E) Statistical analysis of p62 expression (D) and LC3-II conversion (E) From the Western blot (C). Abbreviations: WT, wild type; KO, knockout; MOI, multiplicity of infection; hpi, hours post-infection; N, nucleocapsid.

Autophagic flux can be tracked with the dual-fluorescent RFP-GFP-LC3 reporter [41,42]. WT, VPS33A KO, and VPS18 KO Vero cells were transfected with the RFP-GFP-LC3 reporter. In uninfected WT cells, confocal microscopy revealed diffuse yellow fluorescence throughout the cytoplasm, whereas VPS33A KO and VPS18 KO Vero cells exhibited numerous discrete yellow puncta (Figure 3(A)). Upon PEDV infection, WT cells converted the signal to predominantly red puncta with sporadic yellow dots, indicative of productive autolysosome formation. Conversely, VPS33A KO and VPS18 KO Vero cells retained yellow puncta, demonstrating a specific block in autophagosome-lysosome fusion (Figure 3(A,B)). To further validate that VPS33A or VPS18 deficiency impairs autolysosome formation, we performed additional experiments in porcine IPEC-J2 cells. The results were consistent with those obtained in Vero cells, demonstrating that knockout of VPS33A or VPS18 disrupts autolysosome formation (Figure S3C, D).

Western blot results were consistent with the confocal findings. In WT cells, p62 initially increased and then declined at 12 hpi, while the LC3-I to LC3-II conversion gradually rose, indicating that PEDV infection induces complete autophagy (Figure 3(C–E)). By contrast, VPS33A- and VPS18-deficient cells exhibited sustained p62 levels, persistent LC3-II accumulation, and impaired N protein expression, suggesting that disruption of either VPS33A or VPS18 impedes autophagosome-lysosome fusion, stalls autophagic flux, and attenuates PEDV replication (Figure 3(C–E)).

### Integrity of VPS33A contributes to autolysosome maturation and PEDV propagation

**Figure 4.**
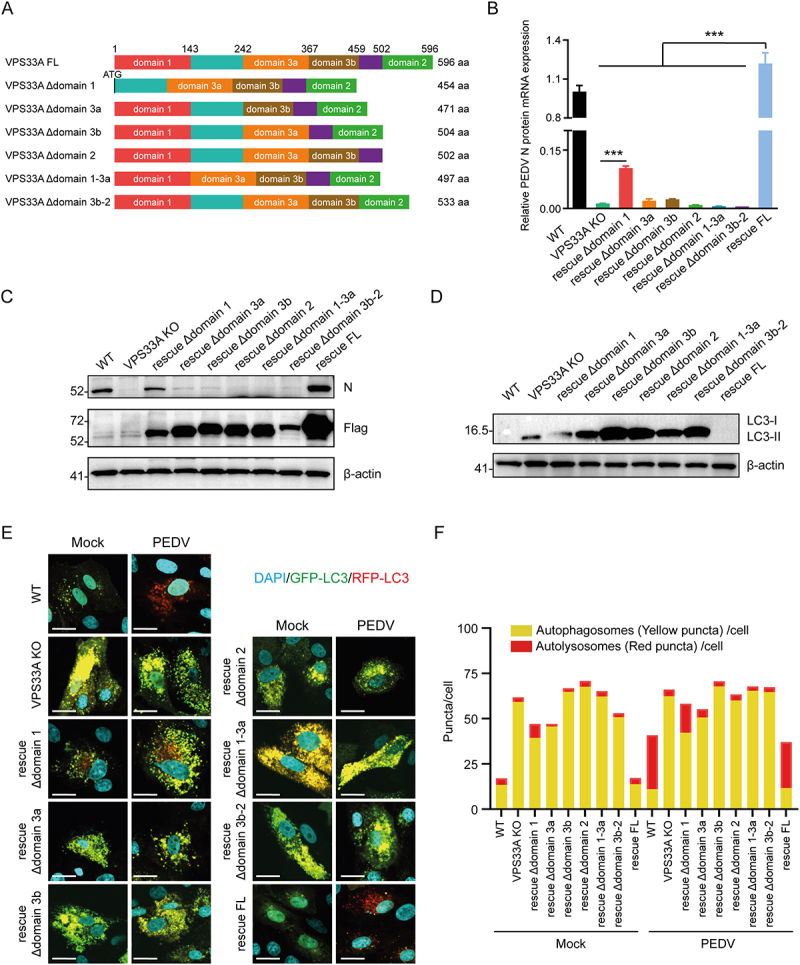
VPS33A integrity contributes to autolysosome formation and PEDV infection. (A) Domain architecture and mutant schematics. (B) RT-qPCR analysis of rescued PEDV replication in VPS33A KO Vero cells reconstituted with the indicated VPS33A mutants. (MOI = 0.01, 24 hpi). (C) Western blot assessment of PEDV N protein recovery under the same conditions. (D) Western blot analysis of LC3 levels in VPS33A KO Vero cells reconstituted with the indicated VPS33A mutants. (E) Confocal images of RFP-GFP-LC3 reporter in uninfected and infected cells (MOI = 0.01, 24 hpi). Scale bars, 20 µm. (F) Quantification of autophagosomes (yellow) and autolysosomes (red) shown in (E). Abbreviations: WT, wild type; KO, knockout; MOI, multiplicity of infection; hpi, hours post-infection; N, nucleocapsid. Data are representative of at least three independent experiments and are presented as mean ± SD. ***p < 0.001. *p*-values were determined by two-sided Student’s t-test.

To map the domains that contributed to autophagosome-lysosome fusion and viral replication, we generated a panel of VPS33A deletion mutants and stably reconstituted them into VPS33A KO cells (Figure 4(A)). After PEDV infection, RT-qPCR and Western blot for the viral N protein showed that only VPS33A full-length (FL) fully restored PEDV replication, whereas reintroduction of any single-domain deletion mutant failed to rescue viral progeny production to WT levels. Intriguingly, the VPS33A ∆domain 1 mutant conferred only a partial rescue (Figure 4(B,C)).

Western blot analysis of LC3 revealed that only re-expression of VPS33A FL fully alleviated LC3-II accumulation compared with WT, indicating restored autophagic flux. The VPS33A ∆domain 1 mutant only partially reduced LC3-II levels, while all other deletion mutants failed to restore autophagic flux (Figure 4(D)). Using transient expression of the RFP-GFP-LC3 tandem reporter, we further observed that VPS33A FL-complemented cells displayed numerous red puncta upon PEDV infection compared to control cells, signifying efficient autolysosome formation. VPS33A ∆domain 1 generated a smaller but detectable number of red puncta, while all other domain deletions yielded only sparse red puncta (Figure 4(E,F)). Taken together, these data demonstrate that the structural integrity of VPS33A contributes to autolysosome formation and optimal PEDV replication.

### VPS18 α-solenoid and RING domains play a role in autolysosome formation and PEDV replication

**Figure 5.**
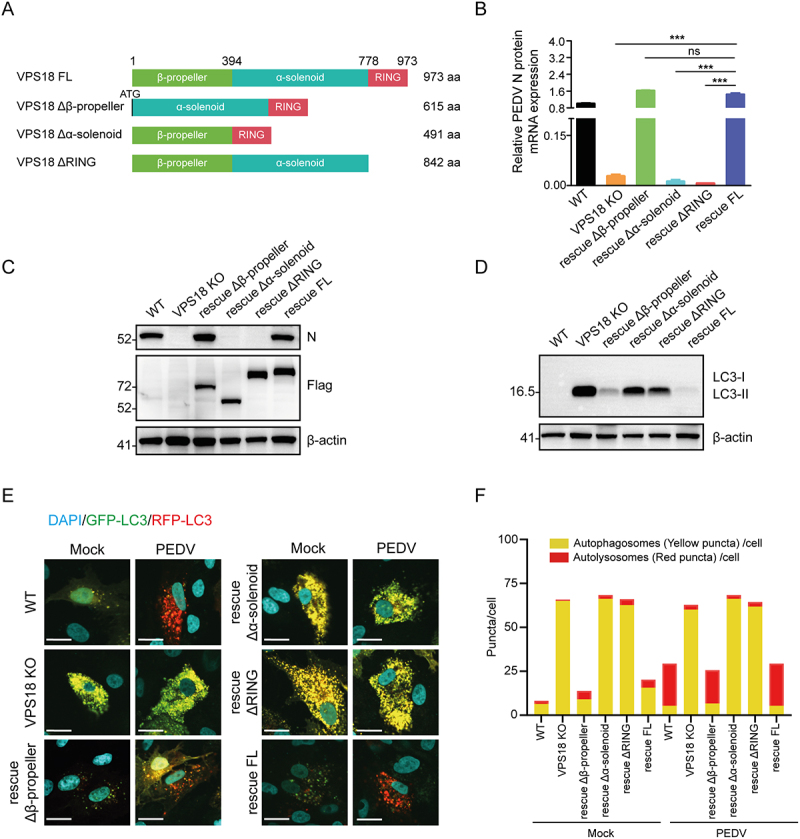
VPS18 α-solenoid and RING domains play a role in autolysosome formation and PEDV infection. (A) Schematic of VPS18 domains and generated mutants. (B) RT-qPCR analysis of rescued PEDV replication in VPS18 KO Vero cells reconstituted with the indicated VPS18 mutants. (MOI = 0.01, 24 hpi). (C) Western blot evaluation of PEDV N protein recovery under the same conditions. (D) Western blot analysis of LC3 levels in VPS18 KO Vero cells reconstituted with the indicated VPS18 mutants. (E) Representative confocal images of RFP-GFP-LC3 reporter in uninfected and infected cells (MOI = 0.01, 24 hpi). Scale bars, 20 µm. (F) Quantification of autophagosomes (yellow) and autolysosomes (red) shown in (E). Abbreviations: WT, wild type; KO, knockout; MOI, multiplicity of infection; hpi, hours post-infection; N, nucleocapsid; ns, no significant. Data are representative of at least three independent experiments and are presented as mean ± SD. ***p < 0.001. *p*-values were determined by two-sided Student’s t-test.

To pinpoint the VPS18 domains that participated in autolysosome formation and PEDV replication, we generated a series of VPS18 deletion mutants and stably re-expressed them in VPS18 KO cells (Figure 5(A)). After PEDV infection, RT-qPCR and Western blot analyses of viral N protein showed that VPS18 FL and the ∆domain β-propeller mutant fully restored PEDV replication, indicating that the α-solenoid and RING domains participate in viral propagation (Figure 5(B,C)).

Consistently, Western blot analysis of LC3 revealed that VPS18 FL and the ∆domain β-propeller promoted LC3-II degradation, reflecting intact autophagic flux, whereas the ∆domain α-solenoid or the ∆domain RING did not (Figure 5(D)). Using the RFP-GFP-LC3 tandem reporter, we observed abundant red puncta, indicative of functional autolysosomes, in cells reconstituted with VPS18 FL or the ∆domain β-propeller upon PEDV infection, similar to WT levels. In contrast, mutants lacking the α-solenoid or RING domain showed only sparse red puncta (Figure 5(E,F)). Collectively, these results demonstrate that the α-solenoid and RING domains of VPS18 play a role in both autolysosome formation and PEDV replication.

### PEDV nsp3 and nsp4 interact with VPS33A/VPS18 and induce complete autophagy

**Figure 6.**
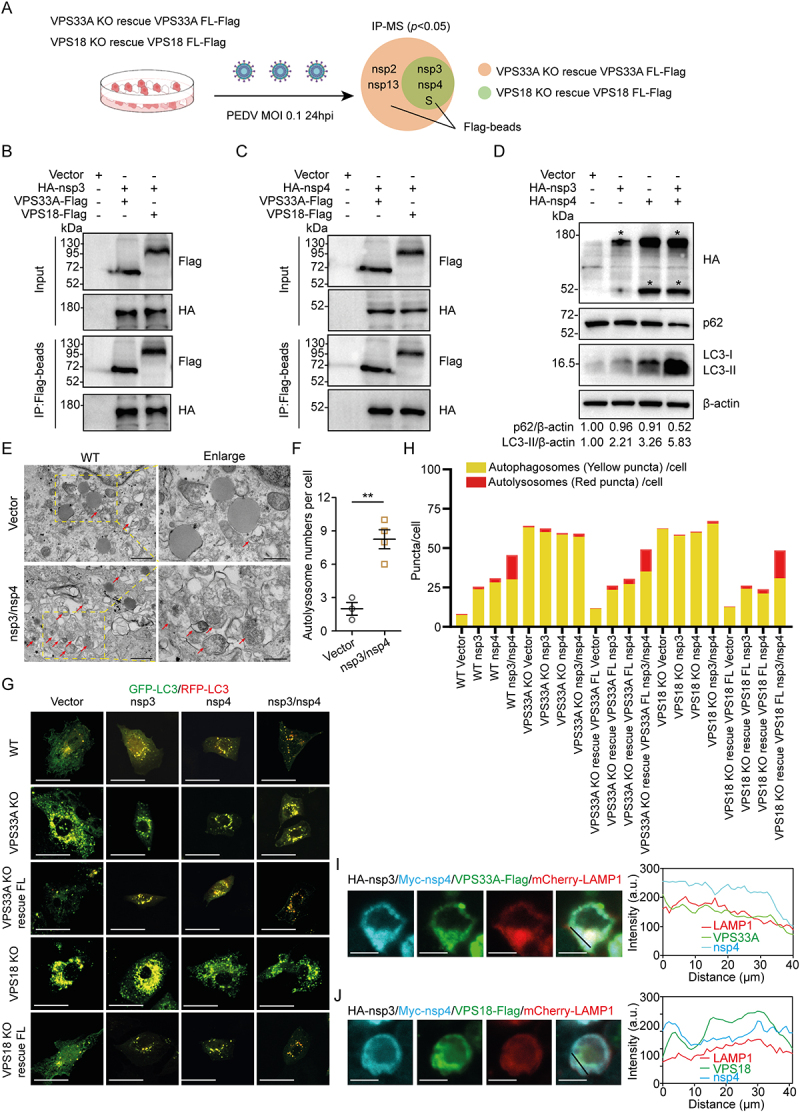
PEDV nsp3 and nsp4 interact with VPS33A/VPS18 and induce complete autophagy. (A) IP-MS identification of viral proteins interacting with VPS33A and VPS18. (B, C) Co-IP confirmation of the interaction between VPS33A/VPS18 and nsp3 (B) or nsp4 (C). (D) Western blot analysis of p62 and LC3 upon transfection with nsp3, nsp4, or nsp3/nsp4. β-actin served as loading control. (E, F) Transmission electron microscopy images (E) and quantification (F) of autolysosomes after co-transfection of nsp3 and nsp4. Scale bars, 1 µm or 500 nm. (G, H) RFP-GFP-LC3 assay images (G) and quantification (H) of autophagosomes (yellow) and autolysosomes (red) under the indicated conditions. Scale bars, 20 µm. (I, J) Confocal fluorescence microscopy analyses of the co-localization of myc-nsp4 (indicated in cyan), VPS33A-Flag (I) or VPS18-Flag (J) (indicated in green), and mCherry-LAMP1 (indicated in red) in HEK293T cells. Measure of the fluorescence intensity of VPS33A or VPS18, nsp4, and LAMP1 at the same location (indicated by the black lines). Scale bars, 10 μm. Abbreviations: WT, wild type; KO, knockout; MOI, multiplicity of infection; hpi, hours post-infection; nsp, non-structural protein. Data are representative of at least three independent experiments and are presented as mean ± SD. **p < 0.01. *p*-values were determined by two-sided Student’s t-test.

To identify viral proteins that interacted with VPS33A/VPS18 and trigger complete autophagy, we reconstituted VPS33A KO and VPS18 KO cells with VPS33A FL-Flag or VPS18 FL-Flag, respectively, and then infected them with PEDV. Immunoprecipitation-mass spectrometry (IP-MS) revealed that both VPS33A and VPS18 interact with nsp3, nsp4, and the S protein (Figure 6(A)). Since depletion of VPS33A or VPS18 did not impair PEDV attachment or internalization but blocked replication, we focused on nsp3 and nsp4. Co-IP further confirmed that both VPS33A and VPS18 interacted with nsp3 and nsp4 (Figure 6(B,C)). Confocal analysis showed that VPS33A and VPS18 colocalized with nsp3 and nsp4, respectively (Figure S4A, B). When Vero cells were individually transfected with nsp3 or nsp4, LC3-II levels rose, yet p62 remained unchanged, indicating that each protein alone elicited incomplete autophagy. Co-transfection of nsp3 and nsp4, however, further elevated LC3-II and markedly reduced p62 compared with either single transfection or control, demonstrating that only the combined expression of nsp3 and nsp4 drove complete autophagic flux (Figure 6(D)). Transmission electron microscopy further revealed that co-expression of nsp3 and nsp4 markedly increased the number of autolysosomes compared with control (Figure 6(E,F)).

Using the RFP-GFP-LC3 tandem reporter, we first observed that single expression of nsp3 or nsp4 in WT cells converted the diffuse LC3 signal into puncta and increased the number of yellow (autophagosome) foci relative to untransfected WT cells, confirming that each protein could initiate autophagy (Figure 6(G)). Co-expression of nsp3 and nsp4 increased both yellow (autophagosomes) and red-only (autolysosomes), indicating that only together were able to induce complete autophagy (Figure 6(G,H)). To test whether this nsp3/nsp4-induced completion of autophagy required an intact HOPS complex, we repeated the assay in VPS33A KO and VPS18 KO cells. Under these conditions, the nsp3/nsp4 pair failed to generate autolysosomes, demonstrating that VPS33A and VPS18 were indispensable for the process. Re-expression of VPS33A FL or VPS18 FL in the respective knockout lines fully restored autolysosome formation only when nsp3 and nsp4 were co-transfected (Figure 6(G,H)). To determine whether nsp3, nsp4, and VPS33A or VPS18 colocalized on autolysosomes (marked by LAMP1), HEK293T cells were co-transfected with nsp3, nsp4, VPS33A or VPS18, and LAMP1. Given that nsp3 and nsp4 themselves can colocalize, and considering the limitations of available fluorescence channels, we ultimately stained for nsp4 as a representative of both nsp3 and nsp4. Confocal microscopy revealed that nsp4 colocalized with VPS33A or VPS18, as well as with LAMP1, suggesting that nsp3 and nsp4 might interact with VPS33A or VPS18 on autolysosomes (Figure 6(I,J)). Thus, nsp3 and nsp4 initiated autophagy individually, but complete autophagy required their simultaneous expression.

## Discussion

PEDV poses a severe threat to swine health, and continuous viral evolution challenges vaccine-based control, underscoring the need to elucidate host-virus interactions for novel drug targets. Here, we generated a genome-wide knockout Vero cell library and identified VPS33A and VPS18 as pro-viral host factors required for PEDV replication. Given the species barrier between swine and African green monkey, these screening results carry inherent limitations and may not fully reflect authentic host factors *in vivo*. Nevertheless, Vero cells remain a classical *in vitro* model for coronavirus studies and provide valuable mechanistic insights.

Autophagy is an evolutionarily conserved catabolic pathway that sequesters cytoplasmic cargo within double-membrane autophagosomes and delivers it to lysosomes for degradation and recycling [43–45]. Viruses have evolved contrasting strategies to manipulate this route. On the antiviral side, DISC1 activates AMPK and suppresses mTOR to restrict Zika virus (ZIKV) replication [46], and TRIM45 upregulates LAMP-2A to accelerate chaperone-mediated autophagic degradation of influenza PB2 [47]. E3 ubiquitin ligase NEDD4 targets PEDV NSP8 for ubiquitination and degradation [48]. Conversely, many viruses convert autophagy into a pro-viral assembly line. For instance, classical swine fever virus (CSFV) hijacks VPS34 complex II to accelerate autophagosome-lysosome fusion [49], and PEDV activates the TAK1-AMPK-JNK axis to drive full autophagic flux that sustains replication, whereas fangchinoline exerts its antiviral effect by blocking this flux [34,50]. However, autophagy also restricts PEDV by promoting BST2/MARCH8/NDP52- and RBM14/p62-dependent selective autophagy of the N protein [51,52]. These studies establish that the functional outcome of autophagy during PEDV infection is context-dependent and shaped by the specific viral proteins and host factors engaged.

The HOPS complex is the central tethering machinery that coordinates late endosome-lysosome fusion and autophagosome-lysosome fusion [14–16]. Its role as a convergence point for membrane trafficking has positioned HOPS as a frequent target during viral infection. SARS-CoV-2 ORF3a binds VPS39, disrupting HOPS-RAB7 interactions and blocking SNARE-mediated fusion to arrest autophagic flux [23,24]. Zika virus likewise targets VPS39 and STX17 to stall autophagosome maturation and hijack accumulated autophagic compartments for replication [53]. African swine fever virus (ASFV) CP204L sequesters VPS39 away from endo-lysosomal membranes to facilitate viral replication [54], and tick-borne encephalitis virus (TBEV) prM binds VPS11, disrupts the VPS18-VPS41 interaction, and constrains autolysosome formation [55]. In contrast to these strategies, our data revealed that PEDV exploits VPS33A and VPS18 to actively promote autolysosome formation, highlighting a fundamentally distinct mechanism of autophagy modulation. Nevertheless, we acknowledge that these conclusions currently rely on HOPS complex perturbation, and independent validation using canonical autophagy genes awaits future investigation.

Our domain-mapping analyses indicated that the structural integrity of VPS33A and specific domains of VPS18 participated in restoring autophagic flux and supporting PEDV replication. However, we acknowledge that loss-of-function phenotypes from domain deletions do not, by themselves, constitute definitive proof of the molecular function of individual domains under physiological conditions. Notably, IP-MS confirmed mutual interactions among VPS33A, VPS18, VPS16, and VPS41. Domain-deletion analyses indicated that domain 1 of VPS33A participated in recruiting VPS16 and VPS41, whereas the VPS18 β-propeller contributed to dispensable for HOPS assembly (data not shown). Consequently, the partial rescue observed with VPS33A Δdomain 1 likely reflects incomplete HOPS reconstitution that permits limited autophagic flux but remains insufficient to fully support PEDV replication, underscoring the stringent requirement for an intact HOPS architecture.

Previous work has shown that PEDV nsp6 and ORF3 trigger autophagy [35–37]. Whether nsp3 and nsp4 modulate autophagy remained unknown. Here we identified nsp3 and nsp4 as determinants of autophagic completion: either protein initiates autophagosome formation, yet their coordinated expression is required for full flux. Both nsp3 and nsp4 physically interacted with VPS33A and VPS18, and their ability to promote autolysosome formation was abolished in VPS33A- or VPS18-deficient cells. Coronavirus nsp3 and nsp4 are core components of double-membrane vesicle (DMV) biogenesis, generating replication organelles that house viral RNA synthesis [56–58]. The N-terminal domain of nsp3 is essential for DMV formation and nsp12 recruitment, whereas the nsp4 C-terminal domain regulates DMV numbers but is not essential for pore assembly [59]. Additionally, coronavirus nsp3 hijacks CLTC to maintain the core class III PI3K complex for autophagosome nucleation, generating precursor membranes that are subsequently hijacked to form DMVs and drive viral replication [60]. DMV membranes are also thought to originate from remodeled ER and share topological similarities with autophagic membranes [61–63]. Our findings raise the possibility that HOPS-dependent autophagic completion and DMV formation are functionally coupled during PEDV infection, with VPS33A and VPS18 potentially coordinating membrane remodeling events required for both autophagy and replication organelle maturation. However, definitive proof of this model requires targeted experiments, which constitute an important future direction.

**Figure 7.**
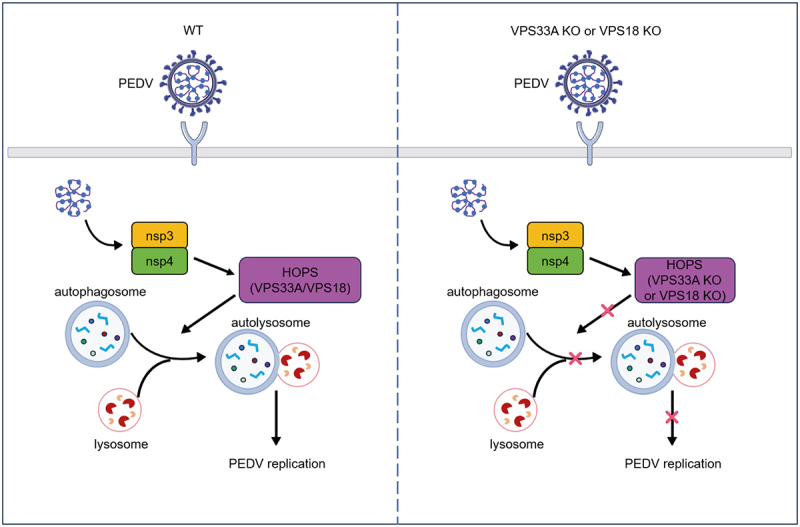
Model diagram illustrating that PEDV nsp3 and nsp4 target the HOPS complex subunits to modulate autophagic flux to promote viral replication.

In summary, our study identifies VPS33A and VPS18 as critical host factors that enable PEDV replication by orchestrating HOPS-mediated autophagosome-lysosome fusion and uncovers a cooperative mechanism by which nsp3 and nsp4 engage the HOPS complex subunits VPS33A and VPS18 to contribute to autophagic completion (Figure 7). These findings establish autolysosome formation as a key host process exploited by PEDV and highlight completion of autophagy as a previously underappreciated determinant of coronavirus replication. By delineating how PEDV co-opts the terminal step of autophagy, our work expands current understanding of virus-autophagy interactions and identifies VPS33A and VPS18 as potential targets for antiviral intervention.

## Materials and methods

### Plasmids and reagents

The sgRNAs were designed using the CHOPCHOP tool. The designed oligonucleotides were synthesized, annealed, and subsequently inserted into the BbsI site of the pCRISPR-sg6 vector. The plasmid pEF1α-Cas9 drives constitutive expression of Cas9 nuclease. The plasmid pVerisprGFP co-expresses the sgRNA together with a surrogate GFP reporter. All sgRNAs used are listed in Table S2.

The HA-nsp3 and HA-nsp4 plasmids were preserved in our lab [13]. The recombinant plasmids pJL-VPS33A-FL, Δdomain 1, Δdomain 2, Δdomain 3a, Δdomain 3b, Δdomain 1-3a, and Δdomain 3b-2-Flag, as well as pJL-VPS18-FL, Δβ-propeller, Δα-solenoid, and ΔRING-Flag, were cloned into the pJL-EF1α-puro and pJL-EF1α-BSD vectors, respectively. The RFP-GFP-LC3 reporter was stored in our laboratory.

Chloroquine (YEASEN, #53755ES60) and rapamycin (YEASEN, #52404ES10) were used in this study.

### Cell culture and electroporation

The Vero (ATCC, #CCL-81) and HEK293T (ATCC, #CRL-11268) cell lines were obtained from American Type Culture Collection (ATCC), and IPEC-J2 (BTCC, #BTCC-4025) from Beijing Bowers Type Culture Collection (BTCC). These cells were routinely maintained in our laboratory and verified to be mycoplasma-negative. They were cultured in Dulbecco’s Modified Eagle’s Medium (Invitrogen) supplemented with 10% fetal bovine serum (Gibco) and 1% penicillin-streptomycin (Gibco), and incubated at 37°C in a humidified incubator with 5% CO_2_.

For transfection via electroporation, we employed the Nucleofector™ 2b Device (Lonza) following the manufacturer’s protocol. Cell-type-specific programs were applied: Y010 for Vero cells and U031 for IPEC-J2 cells. Before electroporation, plasmid DNA was purified to eliminate endotoxin contamination and thereby reduce cytotoxicity, then combined with equilibrated Nucleofector Solution to facilitate effective cellular uptake.

### Viruses

The PEDV CV777 strain (GenBank: AF353511.1) and the PEDV LJX01/GS/2014 strain (GenBank: MK252703.1) were obtained from Prof. Guangliang Liu at the Lanzhou Veterinary Research Institute, Chinese Academy of Agricultural Sciences. The PEDV LJX01/GS/2014 strain belongs to the GII subtype [1]. The PDCoV HeN17 strain (GenBank: OR230676.1) was supplied by Dr. Kunli Zhang from the Institute of Animal Health, Guangdong Academy of Agricultural Sciences.

### Plasmid library construction

To ensure species compatibility with the Vero cell line (derived from the African green monkey, Chlorocebus sabaeus), a genome-scale CRISPR/Cas9 knockout library was custom-designed against the Chlorocebus sabaeus ChlSab1 genome assembly (Ensembl database). A total of 120,139 sgRNAs targeting protein-coding and non-coding RNA genes were selected, along with 2,000 non-targeting control sgRNAs. Following synthesis, the sgRNA pool was amplified by PCR and cloned into the lentiCRISPR v2 backbone (Addgene, #52961) via Gibson Assembly (NEB) at the BsmBI restriction site. The resulting constructs were transformed into DH10B electrocompetent cells by electroporation, and the library was amplified by large-scale plasmid purification using the Endo-Free Plasmid Maxi Kit (Qiagen). Library quality and representation were verified by next-generation sequencing.

### Lentivirus library production

To generate the VeroKOLib lentiviral pool, 10 μg of library plasmid was packaged using 5 μg pMD2.G (Addgene, #12259) and 10 μg psPAX2 (Addgene, #12260) in HEK293T cells that were cultured in 100-mm dishes and transfected with LentiFit reagent (HANBIO, #HB-LLF-1000) following the manufacturer’s instructions. Viral supernatants were harvested at 48 and 72 h post-transfection, passed through 0.45 μm low-protein binding filters (Millipore, #HAWP04700) to eliminate cellular debris, divided into aliquots, and preserved at −80°C.

### Cell library production and PEDV screening

VeroKOLib cell libraries were established by transducing 5 × 10^7^ Vero cells with genome-scale sgRNA lentiviruses at MOI of 0.3 in medium containing 10 μg/mL polybrene (Sigma-Aldrich, #TR-1003). Following 3 days of puromycin selection to deplete non-transduced cells, viable cells were expanded in 100-mm dishes. Deep sequencing confirmed 87.55% sgRNA coverage and 99.13% gene coverage in the resulting libraries. For CRISPR screens, libraries were partitioned into ~3 × 10^7^ cell aliquots, with one serving as a negative control and three replicates designated for screening.

For PEDV challenge, VeroKOLib cells were inoculated at MOI of 0.001 in DMEM/10 μg/mL trypsin and incubated at 37°C, 5% CO_2_. After 2 h, the inoculum was replaced with DMEM/2% FBS. At 60 h post-infection, resistant populations were collected and expanded for subsequent sgRNA sequencing.

### Illumina sequencing of sgRnas in VeroKOLib cells

Genomic DNA was isolated from viable VeroKOLib cells using the FastPure Cell DNA Isolation Mini Kit (Vazyme, #7E0402E4). Targeted amplification of the sgRNA cassette was performed via PCR with Q5 Hot Start High-Fidelity DNA Polymerase (NEB, #M0493). After cleanup, amplicons were subjected to high-throughput sequencing on the Illumina HiSeq TM4000 platform. Gene ranking was subsequently performed based on RRA scores calculated by the RRA algorithm implemented in MAGeCK, with graphical representation of the resulting data. Primer sequences are provided in Table S2.

### Generation of candidate gene knockout pooled and monoclonal cell lines

A dual-plasmid system was employed to generate pooled and monoclonal knockout cell lines. The first plasmid pEF1α-Cas9 drives constitutive expression of Cas9 nuclease, whereas the second plasmid pVerisprGFP co-expresses the sgRNA together with a surrogate GFP reporter. pVerisprGFP was designed and optimized in-house to enable enrichment of on-target cells [64]. Specifically, a 23-nt sequence encompassing the sgRNA protospacer-adjacent motif (PAM) was inserted into the GFP open reading frame, introducing a frameshift that abolishes fluorescence. We previously demonstrated that Cas9-mediated cleavage at the sgRNA-PAM site within the plasmid restores the GFP reading frame, concomitantly inducing genomic editing at the corresponding locus. Consequently, GFP-positive cells were isolated by fluorescence-activated cell sorting (FACS) to enhance the overall editing efficiency of the pooled lines. The monoclonal cell lines harboring homozygous deletions of VPS33A or VPS18 were isolated from the corresponding pooled knockout cell lines. The sgRNA sequences and genotyping primers for the candidate genes are listed in Table S2.

### RT-qPCR

Cellular and viral RNA was isolated using TRIzol Reagent (Invitrogen, #15596018CN) and reverse-transcribed into cDNA with TransScript® II All-in-One First-Strand cDNA Synthesis SuperMix for qPCR (Transgen, #AH341-01) in 10 μL reactions. Quantitative PCR was performed on the QuantStudio® 3 System using 100 ng cDNA template, 5 nM gene-specific primers, and SYBR Green Master Mix (Takara, #CN830A). Thermal cycling conditions consisted of an initial denaturation at 95°C for 5 min, followed by 40 amplification cycles (95°C for 10 s, 60°C for 32 s). Gene expression was quantified by the comparative 2^(^−ΔΔCt^) method, normalized to the GAPDH internal reference. Primer sequences are provided in Table S2.

### Virus plaque assay

Crystal violet plaque assays were performed as follows: WT and VPS33A or VPS18 KO Vero cells were seeded in 24-well plates and infected with PEDV at different MOIs for 2 h at 37°C with 5% CO_2_. The inoculum was then removed, and cells were overlaid with a mixture of 50% 2 × DMEM, 50% Low Melting Point Agarose, 10% FBS, and 1% penicillin-streptomycin. After incubation for 48 h, the monolayers were fixed with 4% formaldehyde for 1 h and stained with 1% crystal violet.

### Immunofluorescence and confocal assay

Following seeding on glass coverslips, cells underwent fixation (4% paraformaldehyde, 10 min), membrane permeabilization (0.2% Triton X-100, 10 min), and blocking (2% BSA, 1 h at ambient temperature) to minimize background staining. Overnight incubation was performed using mouse monoclonal anti-PEDV N primary antibody (Medgen Labs, #SD-2, 1:250), mouse monoclonal anti-dsRNA antibody (SCICONS, #10010200, 1:1,000), mouse monoclonal anti-Flag antibody (Proteintech, #66008–4-Ig, 1:500), rabbit polyclonal anti-HA antibody (Proteintech, #51064–2-AP, 1:500), or rabbit polyclonal anti-Myc antibody (Proteintech, #16286–1-AP, 1:500) at 4°C. After washing, bound antibodies were detected with Alexa Fluor 555 donkey anti-mouse IgG (H+L) (Beyotime, #A0460, 1:500), or Alexa Fluor 488 donkey anti-mouse IgG (H+L) secondary antibody (Beyotime, #A0428, 1:500), Alexa Fluor 405 donkey anti-rabbit IgG (H+L) secondary antibody (Beyotime, #A0605, 1:500), or Alexa Fluor 488 donkey anti-rabbit IgG (H+L) secondary antibody (Beyotime, #A0423, 1:500) for 1 h at room temperature. Nuclear counterstaining was achieved with DAPI (10 μg/mL, 5 min). Image acquisition was conducted on Thermo Fisher Scientific EVOS M5000 or OLYMPUS FV3000.

### Virus titers

To quantify viral yields, WT and VPS33A or VPS18 KO Vero cells were seeded into 12-well plates to form confluent monolayers prior to PEDV challenge at MOI of 0.01. After 24 hpi, culture supernatants were collected and virus titers were measured by TCID_50_ assays using Vero cell lines.

### Western blot

The molecular weight markers used were Share-Bio (#SB-26616) and Vazyme (#MP102) for different experiments. Protein lysates were quantified by BCA assay (Thermo Fisher Scientific, #A55860), with 20–30 μg per lane resolved by SDS-PAGE and electroblotted onto PVDF membranes. After blocking, membranes were probed overnight at 4°C with the following primary antibodies: mouse anti-PEDV N (Medgen Labs, #SD-2, 1:1000), rabbit anti-VPS33A (Proteintech, #16896–1-AP, 1:1000), rabbit anti-VPS18 (Proteintech, #10901–1-AP, 1:1000), rabbit anti-Flag (Proteintech, #20543–1-AP, 1:2000), mouse anti-β-actin (Proteintech, #66009–1-Ig, 1:3000), rabbit anti-p62 (Cell Signaling Technology, #5114, 1:1000), rabbit anti-LC3 (Cell Signaling Technology, #3868, 1:1000), and rabbit anti-HA tag (Proteintech, #51064–2-AP, 1:2000). HRP-conjugated goat anti-mouse (Proteintech, #SA00001-1, 1:3000) or anti-rabbit (Proteintech, #SA00001-2, 1:3000) secondary antibodies were applied for 1 h at room temperature. Signal detection was performed on the Tanon 5200 system, with β-actin serving as the loading control for normalization.

### Viral adsorption, internalization, and replication

WT and VPS33A or VPS18 KO Vero cells were seeded into 12-well plates and grown to ~80% confluence prior to infection assays. Adsorption assay: Cells were challenged with virus at MOI of 10 and maintained at 4°C for 2 h to facilitate surface binding while preventing entry. After three rinses with ice-cold PBS to eliminate non-adherent virions, viral gRNA abundance was assessed by RT-qPCR. Internalization assay: Following virus binding (MOI 5, 4°C, 2 h) and PBS washes as above, cultures were shifted to 37°C for 1 h to permit viral entry. Surface-bound but non-internalized particles were then stripped by three washes with pH 1.5 phosphate buffer, followed by three PBS rinses. Intracellular gRNA levels were subsequently quantified by RT-qPCR. Replication assay: Cells were exposed to virus at MOI of 0.01 for 2 h, washed thrice with PBS to remove residual inoculum, and maintained in DMEM at 37°C for 24 h. After incubation, intracellular gRNA was measured by RT-qPCR as an indicator of viral replication efficiency.

### Immunoprecipitation-mass spectrometry assay

Cells grown to 80% confluency in 10-cm dishes were infected with PEDV at MOI of 0.1. After 24 h the monolayers were lysed on ice for 30 min with NP-40 buffer supplemented with protease and phosphatase inhibitors. After centrifugation (12,000 × g, 15 min, 4°C), the clarified lysates were rotated overnight at 4°C with the anti-Flag magnetic beads (Beyotime, P2115). The beads were collected and washed five times with 1 × TBST, transferred to low-retention tubes, and delivered on ice to the Biological Mass Spectrometry Laboratory, College of Biological Sciences, China Agricultural University, for LC-MS/MS analysis.

### Co-immunoprecipitation assay

To verify protein-protein interactions, transfected HEK293T cells were harvested 24 h post-transfection, washed twice with ice-cold PBS, and lysed on ice for 30 min in NP-40 lysis buffer supplemented with protease and phosphatase inhibitors. Clarified extracts obtained by centrifugation (12,000 × g, 15 min, 4°C) were incubated with anti-Flag magnetic beads (Beyotime, P2115) under constant rotation at 4°C overnight. Following triple washes with lysis buffer, bead-associated complexes were denatured by boiling (100°C, 5 min) in 1× SDS-PAGE loading buffer. Eluates were resolved by SDS-PAGE and probed by Western blotting for target detection.

### Transmission electron microscopy assay

Vero cells were transfected with empty vector or nsp3/nsp4 for 24–36 h, washed three times with ice-cold PBS, and subjected to sequential fixation: initial treatment with 2.5% glutaraldehyde (Servicebio, #G1102) at room temperature (30 min), followed by overnight stabilization at 4°C. Samples were subsequently processed for negative-stain transmission electron microscopy analysis by Scientific Research N Power. High-resolution images were acquired with a HITACHI HT7700 electron microscope.

### Statistical analysis

Significance was assessed using two-tailed Student’s t-tests, with *p < 0.05; **p < 0.01; ***p < 0.001. All experiments were independently replicated a minimum of three times, with representative data presented.

## Abbreviations

Co-IP: co-immunoprecipitation
CQ: chloroquine
DAPI: 4′,6-diamidino-2-phenylindole
DMV: double-membrane vesicle
FL: full-length
HOPS: homotypic fusion and protein-sorting
hpi: hours post-infection
IP-MS: immunoprecipitation-mass spectrometry
KO: knockout
MOI: multiplicity of infection
N protein: nucleocapsid protein
nsp: non-structural protein
ORF: open reading frame
PEDV: porcine epidemic diarrhea viru
Rapa: rapamycin
sgRNAs: single-guide RNAs
TCID50: 50% tissue culture infectious dose
VPS: vacuolar protein sorting-associated protein
WT: wild type

## Supplementary Material

FigureS3.tif

Table S2.xlsx

supplementary figures and tables legends.docx

FigureS4.tif

Table S1.xlsx

FigureS1.tif

FigureS2.tif

## Data Availability

The data supporting the findings of this study and supplementary materials are available in Mendeley (https://data.mendeley.com) under DOI: 10.17632/v4zbbbsr5t.5 [65].
