# Supplementary figures and images for: VPS33A and VPS18 orchestrate porcine epidemic diarrhea virus replication by modulating autophagic flux
Source: Virulence. 2026 Jul 31;17(1):2707880. doi: 10.1080/21505594.2026.2707880 (PMC13432860; doi:10.1080/21505594.2026.2707880)

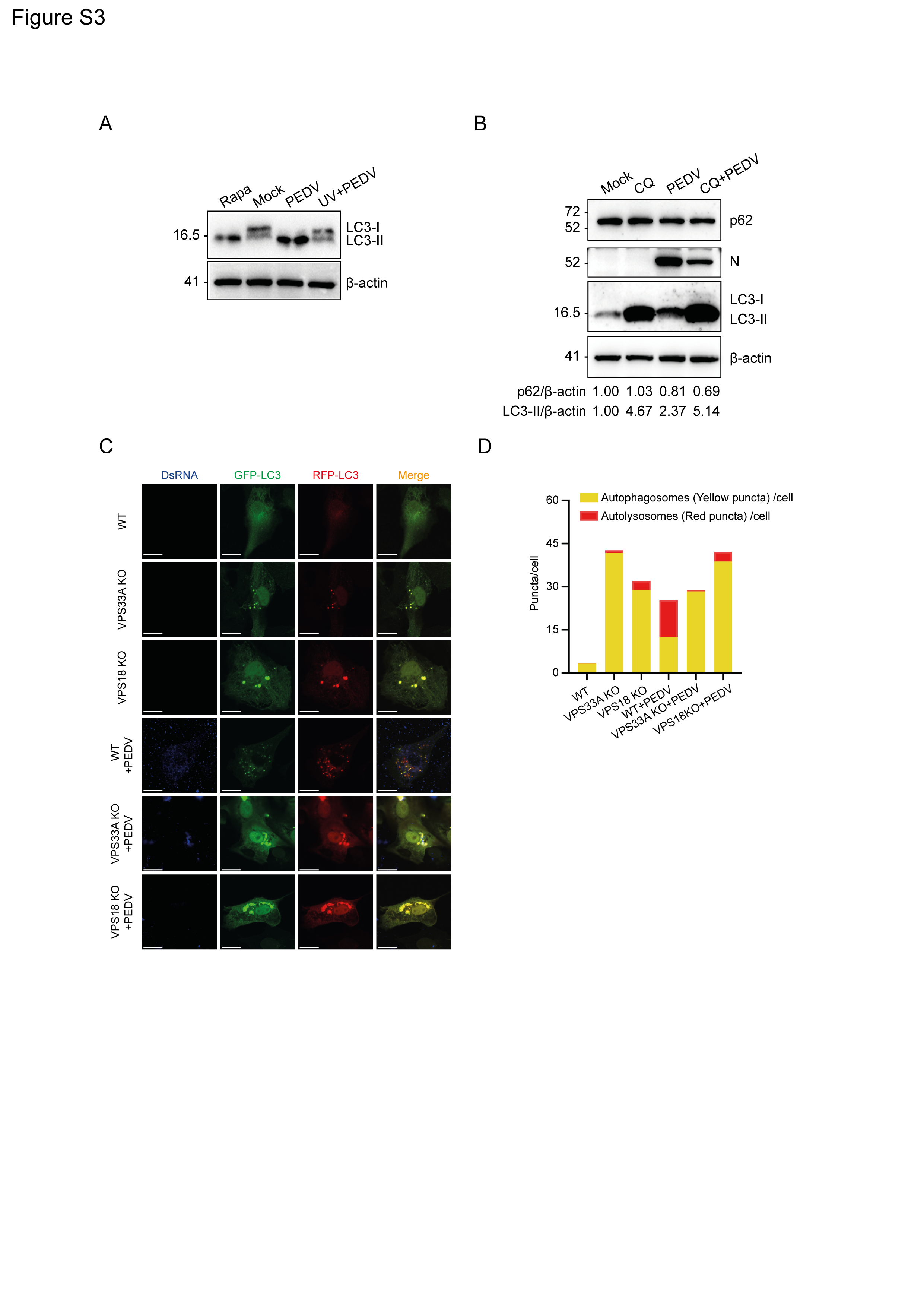

Supplement: FigureS3.tif [file KVIR_A_2707880_SM1630.tif]

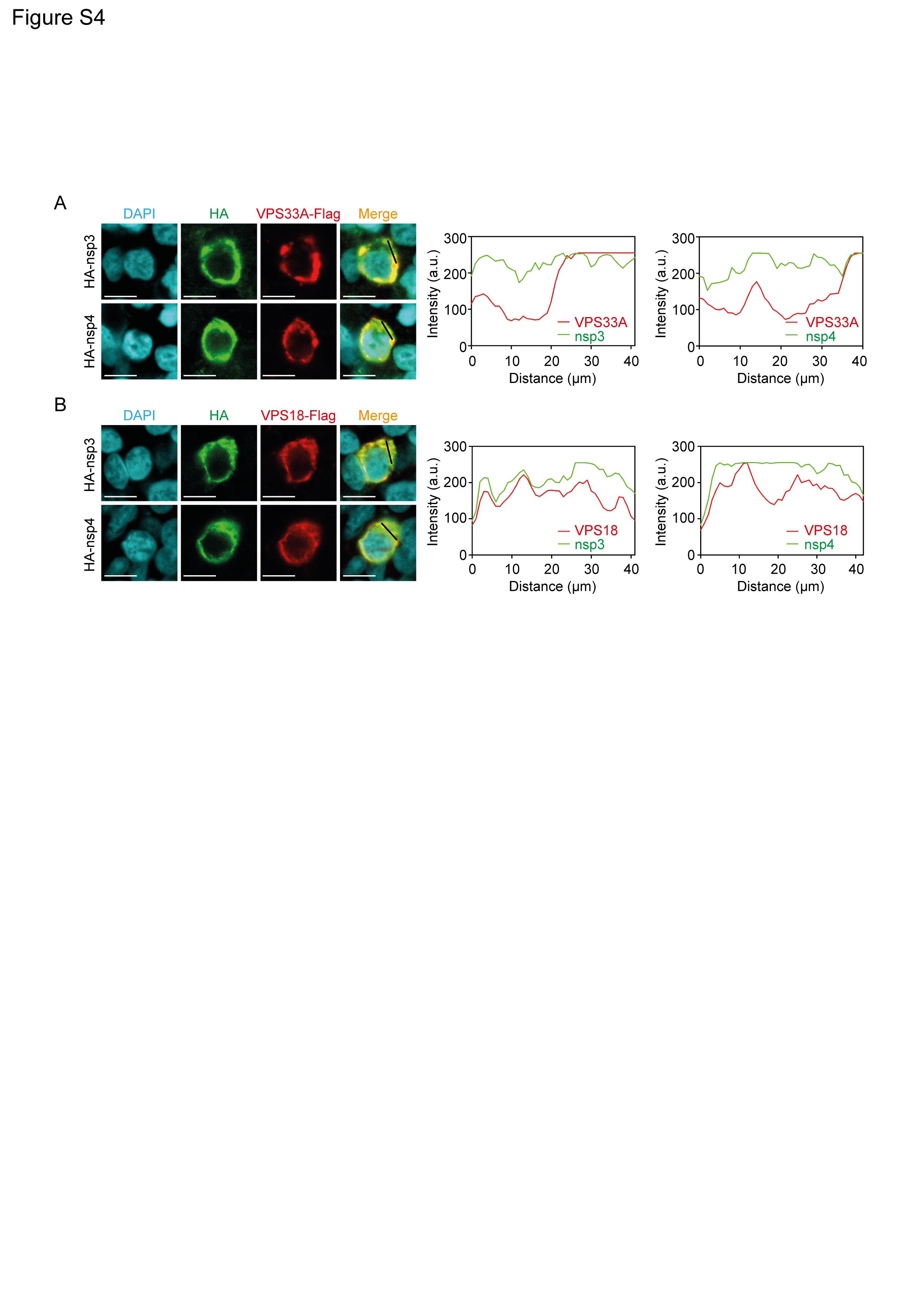

Supplement: FigureS4.tif [file KVIR_A_2707880_SM1627.tif]

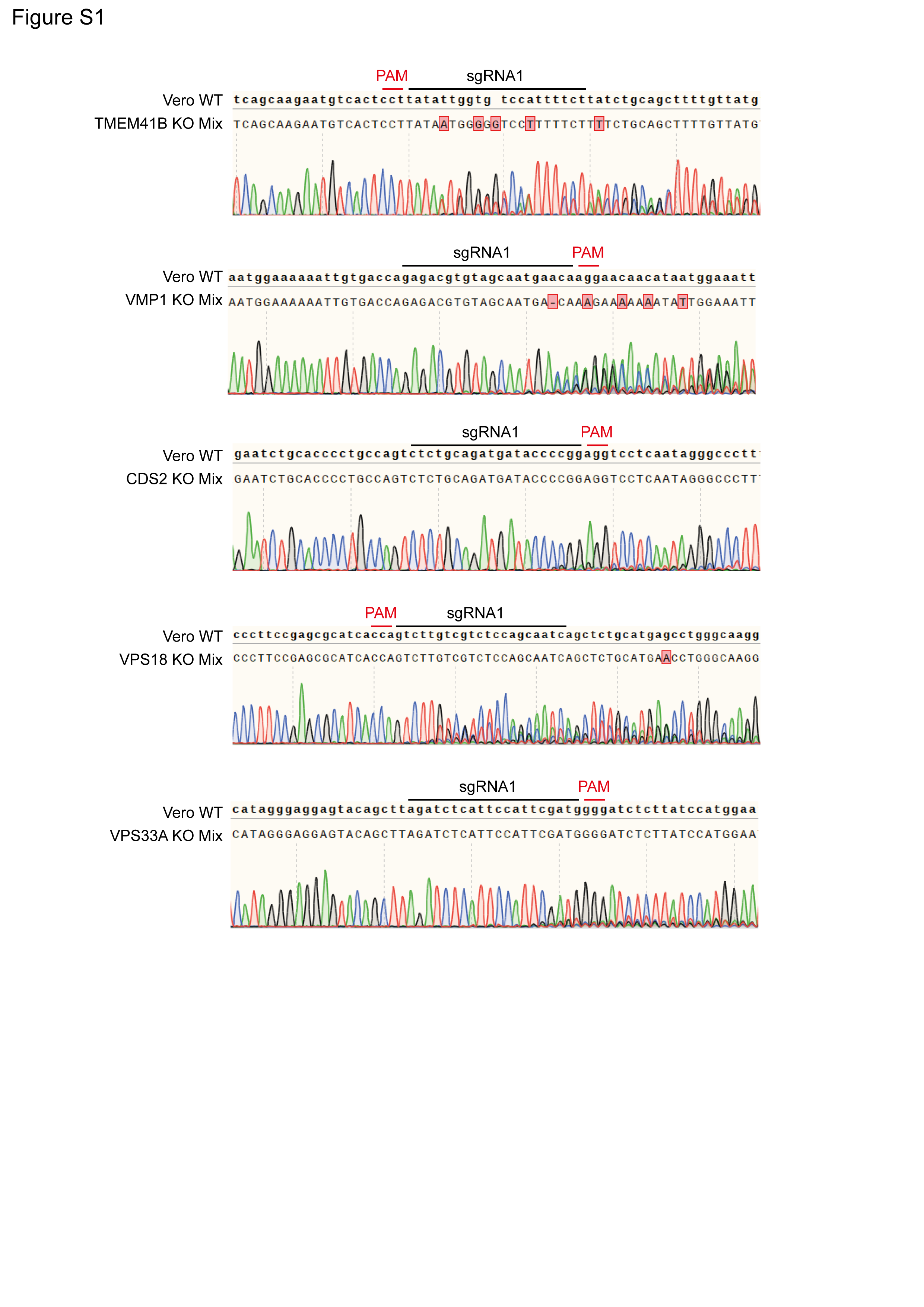

Supplement: FigureS1.tif [file KVIR_A_2707880_SM1625.tif]

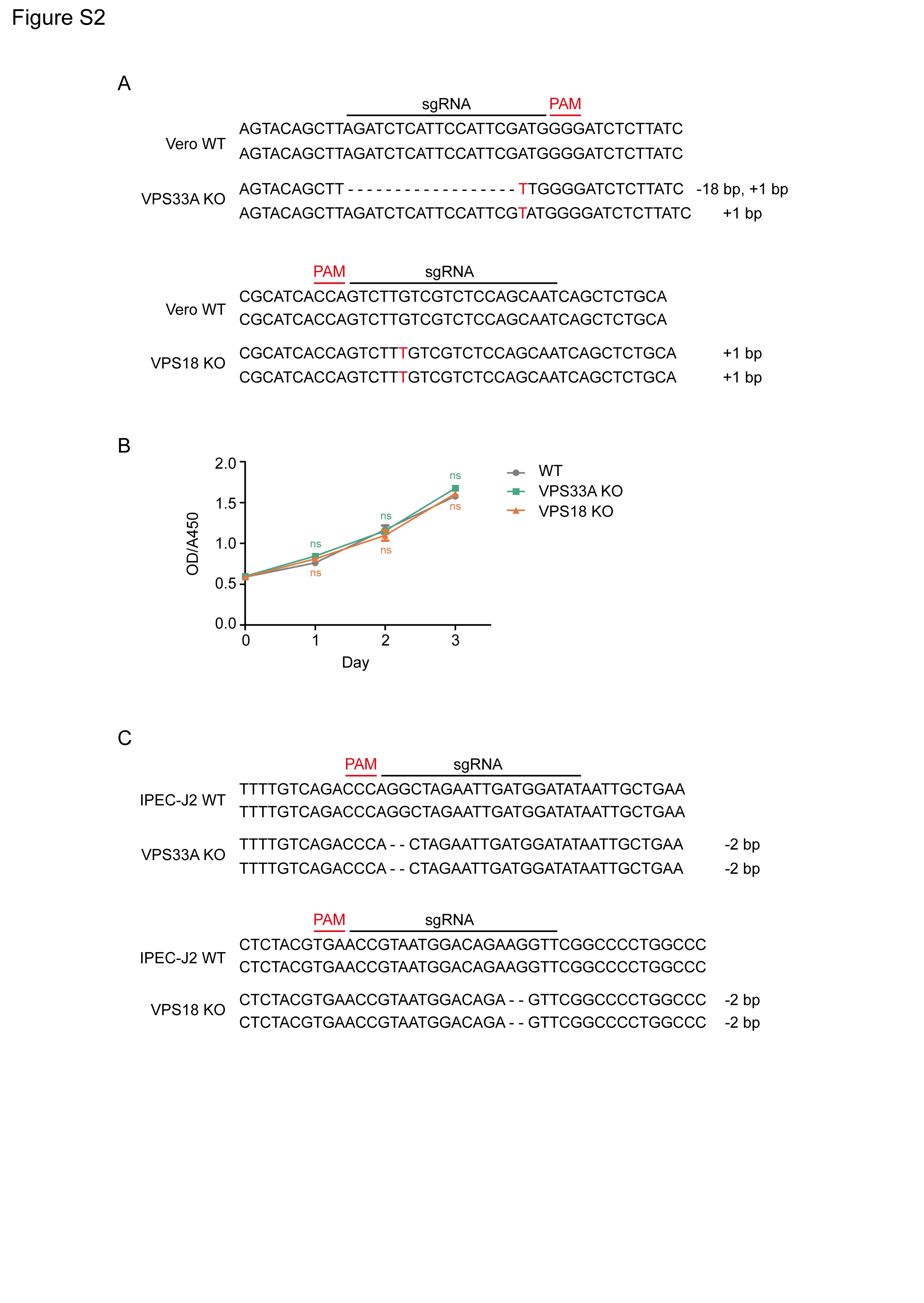

Supplement: FigureS2.tif [file KVIR_A_2707880_SM1624.tif]
