# Supplementary material for: VPS33A and VPS18 orchestrate porcine epidemic diarrhea virus replication by modulating autophagic flux
Source: Virulence. 2026 Jul 31;17(1):2707880. doi: 10.1080/21505594.2026.2707880 (PMC13432860; doi:10.1080/21505594.2026.2707880)
Supplement: supplementary figures and tables legends.docx [file KVIR_A_2707880_SM1628.docx]

**Supporting information**

Figure S1. Presentation of Sanger sequences in the pooled knockout cell library. The black line indicates the sgRNA. The red line indicates the PAM sites. Abbreviations: sgRNA, small guide RNA; PAM, protospacer adjacent motif; WT, wild type; KO, knockout.

Figure S2. Presentation of Sanger sequences in monoclonal knockout cells and proliferation of WT, VPS33A KO, and VPS18 KO cells. (A) Sanger sequences in VPS33A and VPS18 monoclonal knockout Vero cells. (B) WT, VPS33A KO, and VPS18 KO cells were seeded into 96-well plates to evaluate cell proliferation by CCK8 assays at 0 d, 1 d, 2 d, and 3 d. (C) Sanger sequences in VPS33A and VPS18 monoclonal knockout IPEC-J2 cells. The underline indicates the deleted bases in the KO cells. The black line indicates the sgRNA. The red line indicates the PAM sites. Abbreviations: sgRNA, small guide RNA; PAM, protospacer adjacent motif; WT, wild type; KO, knockout; bp, base pairs; ns: no significant. Data are representative of at least three independent experiments and are presented as mean ± SD. *P*-values were determined by two-sided Student’s t-test.

Figure S3. Replication-dependent autophagy in PEDV infection is blocked by chloroquine and requires VPS33A/VPS18 for autolysosome maturation in porcine cells. (A) Western blot analysis of LC3 in Vero cells treated with rapamycin (Rapa), PEDV infection, or UV-irradiated PEDV. (B) Western blot analysis of p62 and LC3 in Vero cells treated with chloroquine (CQ), PEDV infection, or CQ followed by PEDV infection. β-actin served as loading control. (C) Confocal images of autophagosome and autolysosome formation in uninfected and PEDV-infected WT, VPS33A KO, and VPS18 KO IPEC-J2 cells (MOI = 0.1, 48 hpi). Scale bars, 10 µm. (D) Quantification of autophagosomes (yellow) and autolysosomes (red) shown in (C). Abbreviations: Rapa, rapamycin; CQ, chloroquine; WT, wild type; KO, knockout.

Figure S4. VPS33A and VPS18 colocalize with nsp3 and nsp4, respectively. HEK293T cells were co-transfected with VPS33A-Flag/VPS18-Flag and PEDV HA-nsp3/HA-nsp4 after 24 h for confocal assays. (A, B) Confocal fluorescence microscopy analyses of the co-localization of VPS33A-Flag (A) or VPS18-Flag (B) (indicated in red) and HA-nsp3 or HA-nsp4 (indicated in green) in HEK293T cells. Measure of the fluorescence intensity of VPS33A or VPS18 and nsp3 or nsp4 at the same location (indicated by the black lines). Scale bars, 10 μm.

Table S1 Sequencing results of genes and sgRNAs from surviving cells after PEDV infection in the genome-wide CRISPR/Cas9 knockout library.

Table S2 Primer pairs and sgRNA targeting sequences used in this study.
